# The advancement of artificial intelligence in biomedical research and health innovation: challenges and opportunities in emerging economies

**DOI:** 10.1186/s12992-024-01049-5

**Published:** 2024-05-21

**Authors:** Renan Gonçalves Leonel da Silva

**Affiliations:** Health Ethics and Policy Lab, Department of Health Sciences and Technology, ETH Zurich, Hottingerstrasse 10, HOA 17, Zurich, 8092 Switzerland

**Keywords:** Artificial intelligence, Autonomous experimentation systems, Self-driving lab, Research and development (R&D), Drug discovery, Biomedical research, Health, Innovation, Emerging economies

## Abstract

The advancement of artificial intelligence (AI), algorithm optimization and high-throughput experiments has enabled scientists to accelerate the discovery of new chemicals and materials with unprecedented efficiency, resilience and precision. Over the recent years, the so-called autonomous experimentation (AE) systems are featured as key AI innovation to enhance and accelerate research and development (R&D). Also known as self-driving laboratories or materials acceleration platforms, AE systems are digital platforms capable of running a large number of experiments autonomously. Those systems are rapidly impacting biomedical research and clinical innovation, in areas such as drug discovery, nanomedicine, precision oncology, and others. As it is expected that AE will impact healthcare innovation from local to global levels, its implications for science and technology in emerging economies should be examined. By examining the increasing relevance of AE in contemporary R&D activities, this article aims to explore the advancement of artificial intelligence in biomedical research and health innovation, highlighting its implications, challenges and opportunities in emerging economies. AE presents an opportunity for stakeholders from emerging economies to co-produce the global knowledge landscape of AI in health. However, asymmetries in R&D capabilities should be acknowledged since emerging economies suffers from inadequacies and discontinuities in resources and funding. The establishment of decentralized AE infrastructures could support stakeholders to overcome local restrictions and opens venues for more culturally diverse, equitable, and trustworthy development of AI in health-related R&D through meaningful partnerships and engagement. Collaborations with innovators from emerging economies could facilitate anticipation of fiscal pressures in science and technology policies, obsolescence of knowledge infrastructures, ethical and regulatory policy lag, and other issues present in the Global South. Also, improving cultural and geographical representativeness of AE contributes to foster the diffusion and acceptance of AI in health-related R&D worldwide. Institutional preparedness is critical and could enable stakeholders to navigate opportunities of AI in biomedical research and health innovation in the coming years.

## Background

In January 2023, news reverberated across media outlets dedicated to breakthroughs innovations in biotechnology and in the healthcare sector. It announced the initiation of clinical trials for a protein kinase inhibitor INS018_055 – the first anti-fibrotic small molecule inhibitor with promising anti-tumor relevance, designed through the assistance of artificial intelligence (AI). INS018_055 was developed by Insilico Medicine, a generative AI-driven clinical-stage biotechnology company. The discovery of INS018_055 was achieved by a team of researchers from Canada, China, and the United States within the span of less than a month, with results published in *Chemical Sciences* [1]. According to a press release from Genetic Engineering & Biotechnology News (2023) the study “applied AlphaFold [an AI program which performs predictions of protein structure developed by DeepMind, a subsidiary of Alphabet] to an end-to-end AI-powered drug discovery platform (Pharma.AI) that includes a biocomputational engine (PandaOmics) and a generative chemistry platform (Chemistry42), to identify a new drug for a novel target for the treatment of the most common form of primary liver cancer, hepatocellular carcinoma.” [2].

The news of INS018_055’s success circulated globally, highlighting it as a promising result of integrating AI in biomedical research and drug discovery. The AI-generated protein illustrates the potential of the so-called autonomous experimentation (AE) systems to enhance and accelerate the discovery of advanced biochemical entities and responsive bionanomaterials of interest in clinical studies and biopharmaceutical industry.

Also known as autonomous laboratories, self-driven laboratories, or materials acceleration platforms, AE systems are digital platforms capable of running a large number of chemical experiments autonomously. AE are assisted by machine learning (ML) and other robust computational tools with a high level of precision, accuracy and resilience. Those systems can perform in days what scientists would take years to achieve, as proven by the example of INS018_055. Instead of manually replicating experiments and trial-and-error activities, AE systems build robust datasets and run experiments without the physical and intellectual limitations of humans. It reduces the risk for subjective interpretations of findings, due to data robustness and ML-driven hypothesis tests [3–5].

Due to its efficiency in accelerating discovery and rationalizing the use of scarce material resources for R&D activity, AE is expected to have a significant impact on biomedical research. Specifically, areas such as chemical engineering and materials sciences, bioengineering and drug discovery, and molecular systems engineering, are propelling a dynamic pipeline of technologies and solutions of interest for the healthcare sector [6–8].

The promise of success for these systems, however, is in the context of increasing optimism about AI. As an expanding landscape of autonomous labs is being negotiated between scientists, industry, policymakers, and society, there is much to consider regarding the social and political dimensions of these technologies. I question how the examination of AE can shed light on a new wave of transformation in the global biomedical knowledge networks, and in which ways scientists, technology developers, science policymakers, and clinicians from emerging economies can overcome challenges to explore opportunities created by AE, and participate in global knowledge networks in this area.

I am not aware of a study addressing implications of AE systems in biomedical research and health innovation with a specific focus on emerging economies. In recent decades, R&D activities in China and India, for example, have produced impact in the global configuration of biomedical knowledge infrastructures, becoming key players in the biotechnology industry, life sciences and biomedicine [9, 10].

By examining the increasing relevance of AE systems in contemporary R&D activities, this Debate article aims to explore the advancement of artificial intelligence in biomedical research and health innovation, highlighting its implications, challenges and opportunities for stakeholders in emerging economies. I reflect on the place occupied by emerging economies in the “AI in health” global innovation landscape, and what should be overcome to enable stakeholders to navigate the opportunities of AE in the current decade.

This Debate article is structured as it follows. Section 1 “Reconfigurations of biomedical knowledge infrastructures” briefly provides context to emerging economies as potential players in R&D in biomedical research and health innovation. Section 2 “Artificial intelligence and autonomous experimentation systems” discuss the emergence of this very recent field, highlighting its importance to scientific discovery of new chemicals and materials with clinical and therapeutical relevance. Section 3 “Autonomous experimentation in biomedical research and development” brings practical applications of AE in R&D activity, highlighting its relevance in Nanomedicine, AI-assisted drug discovery and precision oncology. Section 4 “Autonomous experimentation in emerging economies” explore challenges and opportunities for stakeholders from emerging economies to join AE efforts, to prepare institutions and society to benefit from AI in health-related innovation and research domains. Finally, “Conclusions” claims the increasing relevance of emerging economies in AE due to its growing capabilities in the area. Additionally, improving cultural and geographical representativeness of AE contributes to foster the diffusion and acceptance of AI in health-related R&D worldwide.

## Reconfigurations of biomedical knowledge infrastructures

For decades, computation, AI, machine learning (ML) tools and other digital technologies have contributed to a technical, epistemic, and geographic shift of biomedical knowledge infrastructures internationally. This cultural and historical process has been examined by humanities and social sciences scholars dedicated to the study of the transformations in science, technology and innovation (ST&I) in society [11].

From the 20th century’s post-war period, ST&I policies have increasingly fostered the development of scientific and technological capabilities of the biotechnology and healthcare sector [12]. Originally centred in the United States and Europe, the global infrastructures of knowledge and policies to advance biomedical research expanded significantly towards regions in southeast Asia in the 1990s and in the edge of 2000s [13]. In that period, the accelerated growth of a biotechnology industry was responsible for decentralizing R&D investments worldwide, promoting local knowledge-based competences in emerging economies. This geographical and technological shift transformed biomedical research and health innovation activities into a convergent field interfacing multiple possibilities in biological, scientific, engineering, and quantitative approaches [14].

From 2000s, the growth of computational digital platforms in scientific research promoted a new wave of technical changes in biotechnology theories and tools. New discoveries in biological engineering, genomics, and bionanotechnology emerged. Countries such as China, South Korea, Singapore, India became players in those areas, with unprecedented expansion in investment in basic research by state-funded S&T policies and corporate R&D instruments [15, 16]. These countries navigated the 2000s as critical players in R&D applied to develop biotechnology-related sectors, biopharmaceutical manufacturing, and precision medicine [17, 18].

However, since the mid-2010s, R&D practices in biomedical research have undergone a further technical, scientific, and political shift. The rapid advancement of computing, big data analytics and AI impacted many areas such as bioengineering, systems and synthetic biology, quantitative biology, and digital health. The STEM fields (science, technology, engineering, and mathematics) have led this emerging data-driven/quantitative biomedical research. This “dislocation” of converging research capabilities, technologies, and policies can be framed as a global process with multiple local manifestations [19]. Biomedical research and health innovation were marked by a shift from experimentation-intensive R&D mainly focused on small improvements and exhaustive adaptation of biotechnologies, to AI-driven resilient experiment systems of scientific discovery and hypothesis testing supported by robust human-computer collaborations, moving rapidly towards the automation of laboratory tasks [20].

But despite global, capabilities to develop those complex AI-driven experimentation systems are still centralized in few locations around the world. Scholars have updated this debate claiming that specific innovations could only emerge in certain environments. Analysts concerned with this topic keep emphasizing the role of location-specific factors in R&D internationalization in high-tech fields, and the implications to multinational enterprises in sectors as such healthcare, biotechnology, information technology and others [21].

The advancement of AI into scientific laboratories is opening new possibilities for biomedical knowledge. New AI tools have implications not only in how expert knowledge is produced, tested and validated, but also in how problems and hypothesis are designed in health innovation such as bioengineered devices, synthetic nanoparticle research, responsive biosystems, cancer vaccines, and molecular diagnostics of diseases [22, 23]. In the Sciences, research has shifted to multidisciplinary teams collaborating in a hybrid (physical-digital) manner, with scientists, engineers, computers and automated lab facilities collaborating to address research problems in ways that would have been impossible to conceive just a few years ago [24].

## Artificial intelligence and autonomous experimentation systems

Beyond automating laboratory tasks, AI tools have furthered the development of systems capable of running experiments and, in some cases, research hypotheses autonomously. We have increasing examples of successful projects in which researchers prototype and improve systems to automatize scientific work, as so-called “robot scientists” [25–27], “self-driving labs” [28], “chemputation” systems [29], “materials acceleration platforms” [30], etc. This collection of emerging technologies is referred to as “autonomous experimentation systems” [31].

AE systems has gained attention from scientists and technology developers, as a tool that “combine robotics for automated experiments and data collection, with artificial intelligence systems that use these data to recommend follow-up experiments” [32]. Its growth corresponds with rapid progress in algorithm efficiency, with AE enabling “the extensive computation exploration of chemical space to design new materials” [28]. AE engines presently signal key trends in bioengineering and biomedical research, materials science, and clinical innovation, with scientists from these fields creating intelligent systems to improve the Design-Build-Test-Learn cycle [7]. This “loop” is a critical principle in the engineering of artificial molecular machines, life-like biochemical components, and self-assembled responsive nanomaterials which are in high demand from the chemical, energy, and biopharmaceutical industries [6].

**Fig. 1:**
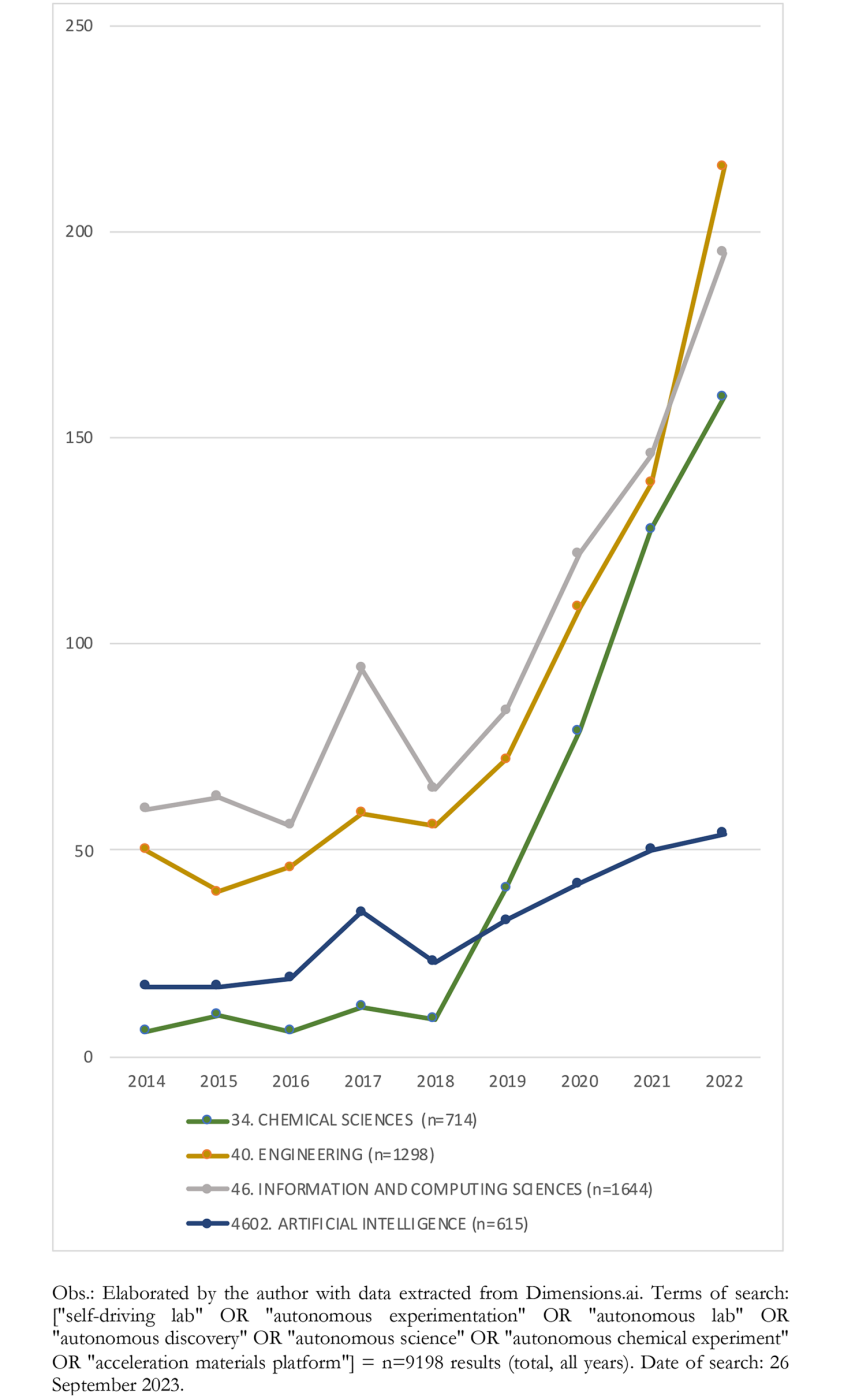
Yearly publications on autonomous experimentation (selected Research Categories), 2014–2022

At present, systems capable of autonomously generating new research hypotheses and chemical combinations are in early stages. References to AE in scientific publications are increasing substantially, with the number of articles between 2018 and 2022 multiplying more than seventeen times for “Chemical Sciences”, four times for “Engineering”, and two times for “Information and Computing Sciences” and “Artificial Intelligence” (see Fig. 1).

## Artificial intelligence in biomedical research and development

Since the creation of the DENDRAL Project, a computer program developed in 1965 by Stanford University scientists to identify chemical compounds, researchers have persevered in the search to automatize chemical experiments using AI [33]. Over the course of decades, the integration of ML, lab automation, and robotics has positioned new data-intensive platforms as fundamental sources of knowledge for facilitating the discovery of novel compounds and materials of biomedical and therapeutic interest. As mature outcomes of this technological development, AE systems such as self-driving labs (SDLs) and materials acceleration platforms (MAPs) can screen thousands of combinations using minimal amounts of starting reagents, enabling the identification of stable compounds with high precision. This has led to increased productivity and efficiency for biomedical exploration of new chemicals and nanomaterials systems, allowing scientists to consider a wider range of solutions to challenging biological problems in a shorter time, impacting the areas of drug discovery acceleration, new materials discovery, and nanomedicine.

**Fig. 2:**
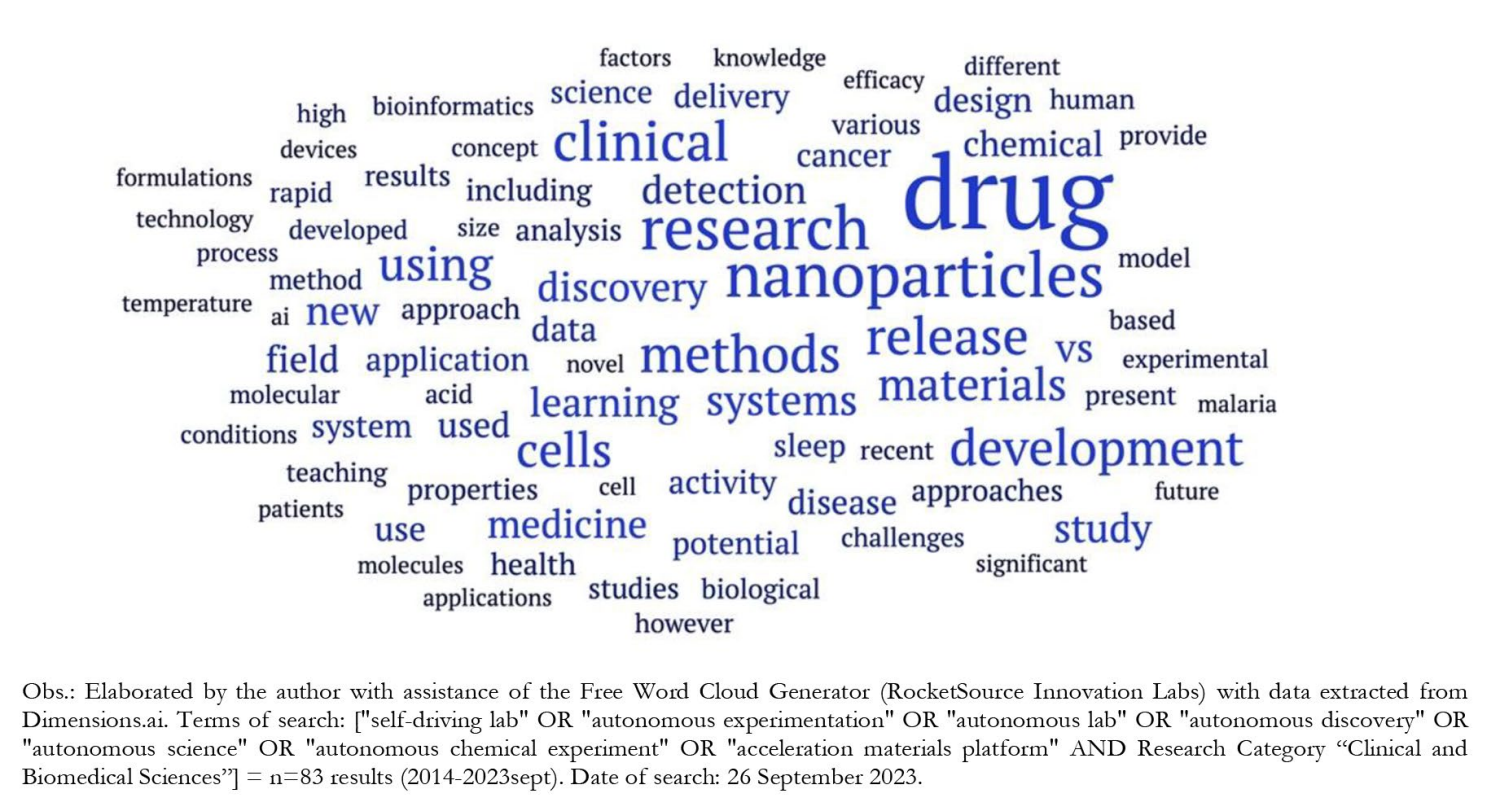
Key words cloud associated to abstracts of publications (*n* = 83) on autonomous experimentation applications in clinical innovation (“Biomedical and Clinical Sciences”), 2014–2023

According to a word cloud generator powered by AI (RocketSource Innovation Labs), using data from 83 abstracts associated with “Biomedical and Clinical Sciences” (2014-September 2023; see Fig. 2), AE clinical applications are mainly related to terms such as “nanoparticles” AND “research”, “materials” AND “development”, “drug” AND “discovery”, “delivery” AND “systems”, and “cancer” AND “detection”. Terms in the cloud indicate some key fields leading the themes related to biomedical research, and the uses of AE in areas as nanomedicine, AI-driven drug discovery, and precision oncology. This three represent relevant research domains in which AE systems have impacted knowledge discovery and technology development of interest to healthcare sector according to the literature. As mentioned above, INS018_55 is an example of area in which the three domains have converged over the last century, i.e., applications of AI in the discovery of nanomaterials of clinical interest and therapeutic function (nanomedicine), AI-assisted drug discovery systems and tools, and generative AI to accelerate discovery of treatments and products in cancer research.”.

### Nanomedicine

The complex nature of nanomedicines is a perpetual challenge to its clinical success. AE has recently produced results with fundamental implications for nanomedicine, employing AI to design nanoparticles with specific properties, optimize drug delivery systems, and predict toxicity, significantly reducing the need for the trial-and-error approach. Automation makes possible the rapid synthesis and characterization of nanomaterials, accelerating the development of novel drug carriers, imaging agents, and therapeutics.

SDLs and MAPs have greatly expedited the discovery and optimization of nanoscale materials for medical use. These platforms employ high-throughput screening techniques and advanced data analytics to assess the properties and performance of thousands of materials simultaneously. As Anselmo and Mitragrotri [34] show, great progress has been made in nanoparticle research over the past five years. The integration of AE in laboratories has accelerated clinical trials of nanocarriers and compounds of therapeutic interest, thanks to innovative approaches for autonomous generation of products [35].

As a result, the development of personalized nanomedicine has become increasingly feasible, offering potential to improve treatment outcomes and reduce side effects. Systems such as the NanoMAP have been proposed to overcome known bioengineering challenges, such as syntheses stabilization and replicability of experiments at nanoscale [36].

AE has recently moved to the forefront of the nanomedicine revolution, allowing researchers to design, synthesize, and test nanomaterials with unprecedented speed and precision. These trends hold great promise for more effective and personalized medical treatments, ultimately benefiting patients and advancing clinical innovation.

### Artificial intelligence-assisted drug discovery

The use of AI in drug discovery has enabled the exploration of vast chemical space, leading to the discovery of novel drug candidates, some of which have already entered clinical trials. The ability to identify promising compounds more efficiently is a game changer for the pharmaceutical industry.

A recent piece in Vox titled “AI-generated drugs will be available sooner than you think” highlighted the availability of many language models applying AI in medicine, and the role of AE in improving the efficiency of R&D, in terms of timelines, costs, and success rates. The author remembers that until the late 2000s, the typical drug discovery process took 12 years, with more than 90% of substances failing in clinical trials [37]. In recent years, AE has harnessed the power of AI and automation to streamline drug discovery processes, significantly reducing time and costs while improving efficiency and accuracy, helping innovators to overcome the so-called ‘Valley of Death’ across preclinical and clinical innovation [38].

A prominent trend in SDLs is the integration of AI-driven robotics and high-throughput screening techniques. By automating tedious and repetitive tasks, AE researchers can focus on more creative and strategic aspects of drug discovery. MAPs, on the other hand, have gained traction in the development of novel drug delivery systems and biomaterials [39].

These platforms have taken drug discovery to a new level, in which techniques can precisely target diseased tissues, release drugs at optimized rates, and minimize side effects, improving patient outcomes. Collaborations between pharmaceutical companies, AI startups, and academic institutions have become increasingly common [40]. As a result, the barriers to entry for smaller companies and research groups have lowered, enabling more widespread adoption of these transformative technologies, with implications for areas such as precision oncology.

### Precision oncology

Recent years have seen remarkable advancements of AI in drug delivery systems discovery for cancer detection and therapeutics, and improving existing systems. The combination of AE systems with robust AI tools is revolutionizing the way researchers approach cancer treatment, offering unprecedented precision, accuracy, and specificity [41].

As AE researchers increasingly adopt AI algorithms to automate drug synthesis and screening, these AI-driven systems can rapidly analyze vast datasets, and design customized drug delivery materials tailored to individual patient profiles. This level of personalization holds immense promise for cancer treatment, with highly targeted therapies that minimize side effects increasingly attainable.

Recent trends in biomedical engineering devices and technologies illustrate the level of technical convergence of contemporary biotechnology research. For example, the use of microfluidics and engineered microphysiological systems (lab-on-a-chip or tissue/organ chips) to predict drug response, and serve as an animal substitute in pre-clinical trials, is growing [42]. These platforms enable precise manipulation of tiny volumes of fluids, making it possible to create and test novel drug delivery systems quickly and efficiently. Those devices mimic the complex biological microenvironments found within tumors, facilitating more realistic in vitro testing of new chemicals and responsive bio nanomaterials, accelerating the discovery of innovative drug delivery systems to navigate the challenges of cancer’s heterogeneous nature.

Due to the large number of biochemical reactions that they enable, AE systems are useful for efficiently screening and optimizing materials for qualities like biocompatibility, drug release kinetics, and targeting specificity, expediting the translation of promising drug delivery systems and reducing the time and cost of bringing new therapies to market [43].

Finally, 3D printing is gaining traction in nanoengineered cancer disease models [44], enabling highly customizable drug delivery vehicles at the nanoscale (by so-called ‘nanocarriers’). AE can design nanoparticles, liposomes, and other carriers with precise control over their size, shape, and surface properties. Such precision is essential for enhancing drug delivery to cancer cells while minimizing harm to healthy tissues [45].

AE underscores the importance of nanoscale materials in the development of next-generation cancer therapies. A combination of precision oncology tools such as AI-driven labs, microfluidics, 3D printing, and nanocarrier engineering are converging to create a powerful synergy to accelerate drug discovery for cancer treatment. As AE and precision oncology continue to advance, the outlook for cancer patients should become increasingly hopeful, with potential for more targeted and less invasive treatments.

## Autonomous experimentation in emerging economies

The examples above demand robust investment in science and technology, to thrive as platforms of biomedical knowledge production and true clinical impact. In this section, I describe what I see as challenges and opportunities for stakeholders from emerging economies to join these efforts, to prepare institutions and society to benefit from AE in biomedical research and health innovation.

### Challenges

**Fig. 3:**
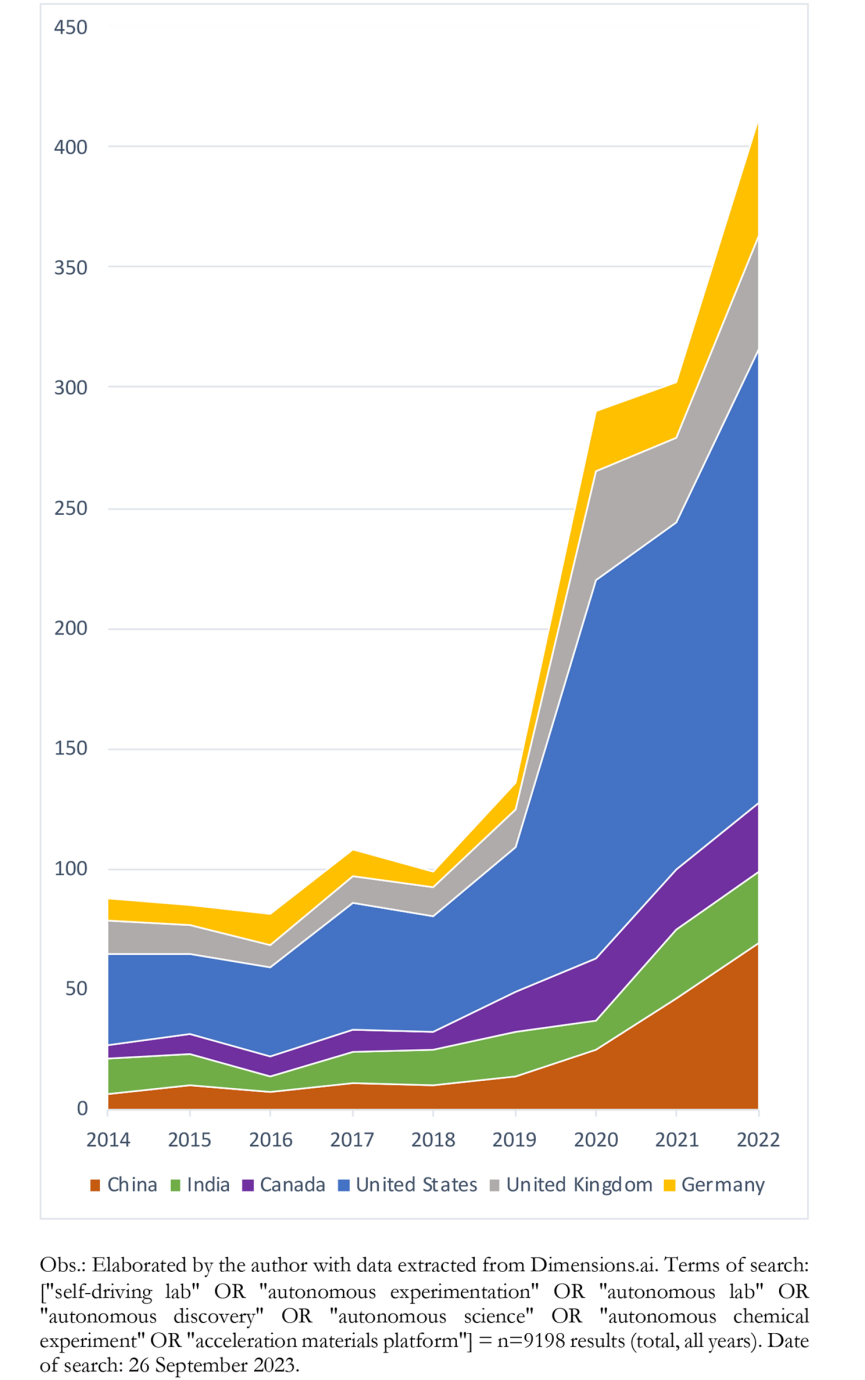
Yearly publications on autonomous experimentation systems, selected countries, 2014–2022

Despite the predicted global impact, AE R&D has historically been concentrated in entrepreneurship in North America and Europe. Projects have been conducted by groups of scientists in developed countries with consolidated science and technology policies and mature national systems of innovation. Figure 3 (supported by data extracted from Dimensions.ai) [46] demonstrates the rapidly growing number of annual publications from the United States, Canada, and Germany. Researchers in China and India have improved their presence in the field significantly, reinforcing the need to examine AE trends beyond North America and Europe.

Below I select six challenges faced by stakeholders from emerging economies seeking to enter the field of AE.

#### Persistent issues in education for science and technology

Performance in AE research is closely linked to a country’s ability to cultivate a national workforce with strong qualifications in the STEM fields. It has implications in how competitive R&D centers are in attracting individuals with exceptional backgrounds in mathematics, programming, and the natural sciences, including professionals from abroad [47]. STEM education is fundamental for training scientists in automation, digitalization, and automatization of biomedical research.

Emerging economies face unique and persistent challenges in Science education, which might lead the research in those countries into a prolonged gap in AE expert knowledge. According to the New York Academy of Sciences’ 2015 report “The Global STEM paradox”, 90% of skilled workers from Caribbean countries leave home to pursue opportunities overseas. Likewise, the World Bank shows that “African countries lose 20,000 skilled professionals to the developed world each year and, as of 2011, one in every nine Africans with a graduate degree lives outside the continent.” [48]. This is not only an issue in places with low levels of economic activity and growth. Even large markets as Brazil struggle as a relevant economy with persistently poor levels of STEM education [49].

However, from the 1990s, we can see a clear trend of emerging economies who have succeeded at fostering STEM fields as a driver of a qualified workforce – being top-ranked in STEM education even when compared with high-income societies. According to the Center of Excellence in Education (CEE) Index of Excellence in STEM Education, China has led the rankings for the last 30 years, with Russia ranked in second place. Students in Taiwan are positioned in fourth place, followed by Singapore, South Korea, Vietnam, Romania, Hong Kong, and Iran [50].

While it is not possible to trace a linear relationship between STEM education and AE initiatives, the index provides some indication of which countries are most likely to advance AI for scientific research enhancement and clinical applications. It can thus inform institutional preparedness and policymaking, towards future AE-assisted innovations in the biomedical sector.

#### Non-resilient science and technology policies

Governments worldwide experience fiscal problems, political tensions, crises, and other inevitable shocks in governance of national policies. These realities affect the resilience of S&T policies, with financial impacts, among others. Extensively studied, resilience is a critical aspect of a well-successful system of S&T policies and initiatives, and is associated with progress and breakthroughs in basic research, innovation and catching-up of knowledge-intensive sectors as the biotechnology and biopharmaceutical industries [51–54]. For example, in comparing S&T policy between the United States and China, scholars note the value of resilience for US basic science research over the long term [55, 56].

**Fig. 4:**
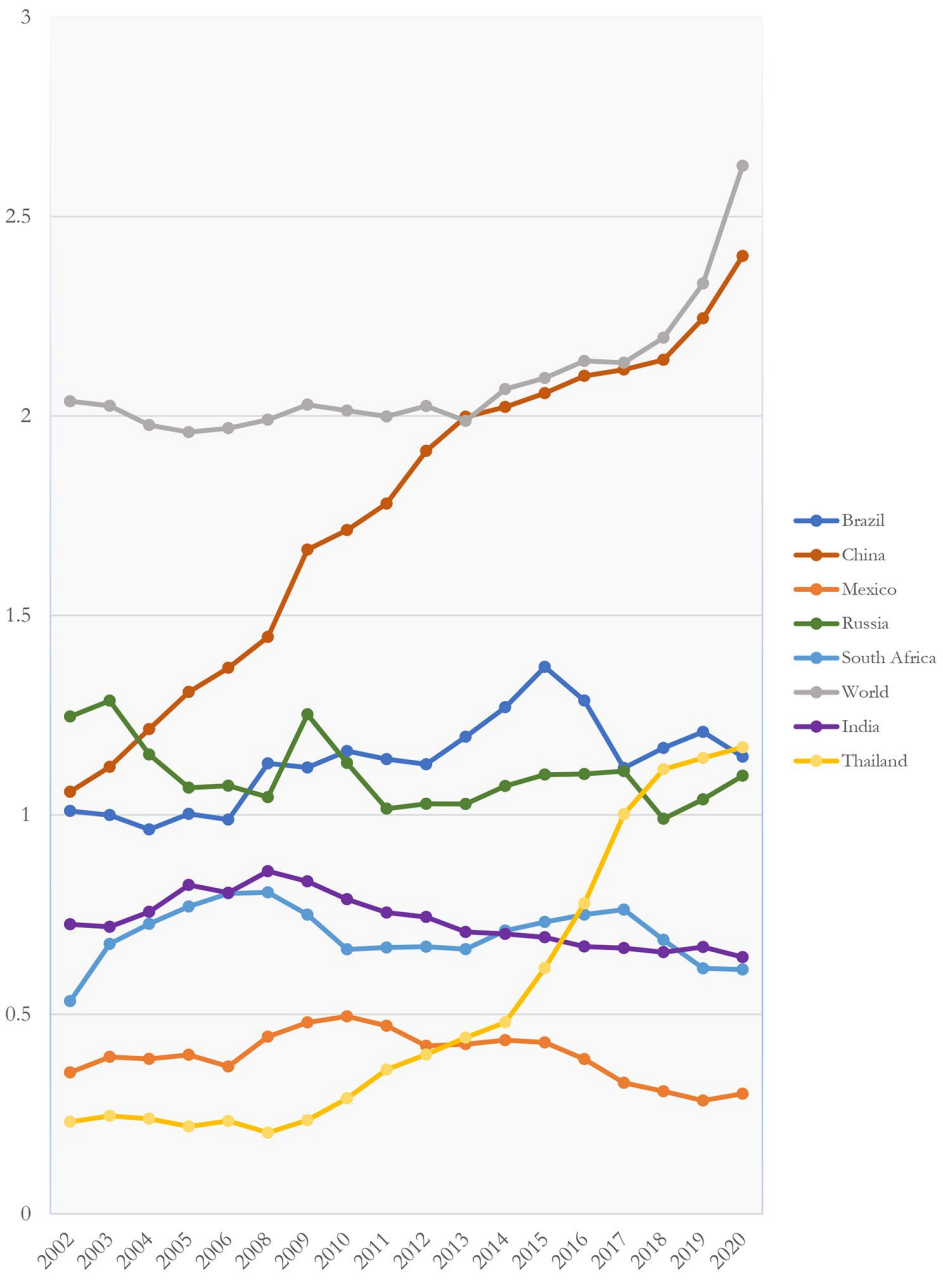
R&D Expenditure (% of GDP), Selected countries and World, 2002–2020. Source: elaborated by the author with data from World Bank, OECD, Statista and National Governments

As Fig. 4 shows, between 2002 and 2020, investment in R&D as a percentage of GDP grew significantly in countries like China and Thailand, but stagnated in countries such as Russia, Brazil, Mexico, and South Africa; S&T innovation did not see substantial growth in these countries during this period (See Fig. 4).

In some emerging economies, despite political and economic crises, S&T policies have resulted in curious paradoxes. For example, the fact that Brazil and India have increased STEM graduates from 4 million to 5 million annually in the second half of the 2000s, while countries such as the United States, United Kingdom, and Japan continued to produce 1 million graduates each year [48].

Considerable effort has been devoted to analyzing investment in applied research and technology transfer within emerging economies [57]. Table 1 illustrates the increasing significance and involvement of funders from China and South Korea, identified as key emerging contributors to the resources allocated for AE R&D, as mentioned by scientists in indexed publications (mainly the National Natural Science Foundation of China and the Ministry of Science and Technology of the People’s Republic of China). However, scientific publications in AE systems are still concentated and focused on its growth in United States and European countries. Agencies of the National Science Foundation and National Institutes of Health in the United States, European Commission (EC), European Research Council (ERC) and the German Research Foundation are also frequently associated with AE publications (Table 1).

**Table 1:** Ranking of 20 funders (number of mentions in indexed publications), 2008–2023

Funder	*n*. mentions in indexed publications
National Natural Science Foundation of China (China)	1378
Ministry of Science and Technology of the People’s Republic of China (China)	395
European Commission (European Union)	173
Ministry of Education of the People’s Republic of China (China)	131
National Research Foundation of Korea (South Korea)	130
China Postdoctoral Science Foundation (China)	119
Chinese Academy of Sciences (China)	117
National Science Foundation - Directorate for Mathematical & Physical Sciences (United States)	111
National Cancer Institute NCI (United States)	103
National Institute of Biomedical Imaging and Bioengineering (United States)	86
Ministry of Education (Japan)	75
Japan Society for the Promotion of Science (Japan)	71
European Research Council (ERC) (European Union)	70
National Institute of General Medical Sciences (China)	70
Ministry of Science and ICT (South Korea)	67
National Science Foundation - Directorate for Engineering (United States)	65
Deutsche Forschungsgemeinschaft (Germany)	62
Fundação para a Ciência e Tecnologia (Portugal)	62
Ministry of Economy, Industry and Competitiveness (Japan)	62
Science and Technology Commission of Shanghai Municipality (China)	61

*Source* Data extracted from Dimensions.ai for the term “Terms of search: [“self-driving lab” OR “autonomous experimentation” OR “autonomous lab” OR “autonomous discovery” OR “autonomous science” OR “autonomous chemical experiment” OR “acceleration materials platform”] (2008–2023). Date of search: 26 September 2023” [46]

As discussed by many scholars, STEM capabilities play a critical role in emerging areas of the so-called “Convergence Sciences” as one could list computer-aided drug design systems [58], computational chemistry [59], AI-informed computational biophysics [60], and others.

This might be an straightforward claim in global technology hubs in the north, with much investment coming from both committed governments and/or private stakeholders [61]. The resilience of S&T policies in high-income countries may be partly attributed to complementary R&D expenditure between the public and private sectors, which supports innovation when economies and governments face crises [62]. However, and as we all know, this is not the reality in the Global South societies. Due to impeditive costs, high failure rates, and resistance to disruptive technologies, AI-enhanced initiatives can require sustained government investment until risks are sufficiently reduced to elicit private sector collaboration and investment.

In fact, investors are now more eager and willing to invest in AI related technologies in emerging economies [63] but much research is needed to know in what sense those investments are building permanent research infrastructures adequate to future integration of stakeholders from emerging economies in the global knowledge and technology networks in AE. Stakeholders from emerging countries should rethink the role of public and private investment in research and how they are actually leading AI initiatives to produce new science and technologies [64]. In addition, universities and research institutes can play a fundamental role in coordinating initiatives and promoting AE institutional preparedness and programs.

#### Competitiveness in attracting global talents

Improving the competitiveness of institutions for attracting international talents is crucial for basic research and technological innovation. In more than a decade studying how scientists conduct their work in public and private laboratories in biochemistry, genomics, biopharmaceutical manufacturing and development, molecular systems engineering, and bionanomaterials discovery, it is easy to recognize the value of internationalization and cultural diversity for science. Successful graduate programs and steady flows of talented and hard-working immigrants are fundamental to support the work of professors and senior scientists, and build research programs, where immigrants regularly become indispensable leaders [65].

Robust internationalization initiatives for graduate programs are one means to better position emerging economies institutions to access global STEM expertise and to be part of AE knowledge and innovation networks. However, internationalization is also dependent on investments done in Education for science and technology. Overcoming persistent issues about educational gaps and brain drain are still relevant, and some emerging countries do it better than others.

While language barriers and lack of resources are regularly used to explain the inability of scientists from emerging economies to access critical STEM research capabilities [66], countries such as South Korea, India, and Singapore have demonstrated that these factors offer only a partial explanation. Institutions from these countries have effectively integrated themselves into global academic networks partially through successful policies for internationalization of graduate and research programs, well-funded by universities, governments and companies [67]. For example, Nanyang Technical University, the Chinese University of Hong Kong, and the Korea Advanced Institute of Science and Technology (KAIST) in Seoul are cases of institutions who have overcome the one-way road of talent departure [68]. This can be viewed as a significant outcome of past investments in R&D capabilities within some emerging economies. Scholars dedicated to the examination of R&D dynamics in late industrialized economies show that, especially for the cases of China and South Korea, investments have led to more productive systems for fostering university-industry links, particularly as their funding mechanisms become more diversified, formalized and stable over time [69].

#### Quality of collaborations in clinical studies

International collaboration in biomedical research is fraught with challenges for emerging economies, often characterized by delayed collaboration in clinical trials. A seemingly simple question has the potential to shed light on the role of global south in large scientific and technological partnerships. This question pertains to areas in which scientists and stakeholders from the low and middle-income countries are specifically sought out for clinical trial collaboration, and why they considered critical to its success [70].

Studies have provided a critique of the nature of clinical trial collaboration between stakeholders from high-income countries and collaborators in emerging economies. Countries like India, Brazil, and some Central American nations have become hubs for clinical trials sponsored by multi-national pharmaceutical companies, who hold exclusive rights to new technologies [71, 72]. If emerging economies serve as crucial testing grounds, contributing considerably to advancing health technologies, questions of fair distribution of benefits arise. For example, to what extent do these collaborations strengthen local scientific expertise? Will global south scientists take an active role in shaping the early stages of technology design of AE systems to enhance knowledge infrastructures in R&D and clinical studies capabilities? These are significant questions for contemporary biotechnology research. In addition, in limited resource settings, the question of whether clinical trial collaborations should be given priority (allocation of funding, human resources) over basic research is an important one to consider.

These questions relate to emerging economies’ “technology sovereignty”. Here I adopt the notion of “technology sovereignty” from the recent work of Jakob Edler and colleagues (2020; 2023), who define it as “the ability of a state or a federation of states to provide the technologies it deems critical for its welfare, competitiveness, and ability to act, and to be able to develop these or source them from other economic areas without one-sided structural dependency.” [73, 74]. Technology sovereignty is critical in AE co-development, to ensure that clinical innovation accelerates while national knowledge capabilities are preserved. Since the Covid-19 crisis, states have been under pressure to develop more resilient and sustainable national infrastructures for health technology development [75, 76].

The integration of AE into health innovation is expected to exert significant pressure on both researchers and industry players. Authorities in emerging economies must proactively build scientific and technological capacities within local universities and healthcare systems to address the growing number of drug candidates generated with assistance of AI entering the market. This preparation will inherently require more rapid and extensive clinical trials and participant recruitment [77], while maintaining high standards of accuracy and compliance with protocols and regulations of pharmaceutical agencies [78, 79].

The great challenge for stakeholders in emerging economies is in leveraging local biomedical infrastructures to capitalize on this emerging trend, overcoming their historic role as knowledge dependent-systems and clinical trial hubs. This shift has potential to propel national innovation systems to transcend the traditional North-South divide in biomedical research.

#### Health systems’ disconnection from R&D activities

Health systems in emerging economies regularly face significant fiscal and political constraints, and many have experienced defunding over the past two decades [80, 81]. This is a challenge not exclusive to global south societes [82]. However, and beyond its institutional mission of offering qualified healthcare services, health systems are important assets for R&D activity and health innovation [83], as well as critical to assist decision-making on relevant national health policies and health technology initiatives and programs [84, 85].

Reliable health systems are key to supporting clinical innovation and access to health technologies. During the Covid-19 pandemic, for example, in countries like China, Brazil, and India, collaborations between scientists, technology developers, and public health systems facilitated development and distribution of locally produced Covid-19 test kits, thanks to ad-hoc coordination between universities, regional science policy instruments, state laboratories, regulators, and health systems [86–88]. Thus, health systems could play a critical role in collecting patient data to support research, and in creating new platforms in the early stages of AE development [89].

When incorporated effectively, health policies can inform national strategies of technology development, and serve as catalysts of sectoral S&T collaboration. Case studies from emerging economies offer valuable insights into the role of healthcare systems, including examples such as:

- Dialogue between health systems and experts that led national authorities to invest in R&D for dengue technologies in the Philippines [90];
- Forging of connections between medical authorities and regional scientific resources to propel a molecular biology-driven cancer research agenda in Brazil, establishing its technical and political feasibility through claims of scientific impact allied with its public health relevance [91];
- Management of knowledge about Ebola through local medical and scientific collaborations in Guinea, Mali, Ghana, and Kenya [92];
- Negotiations within an international consortium of experts on responsible innovation for Zika Virus [93].
- Collaboration between health systems and scientists in China and Brazil to establish platforms for genomic data for use in precision medicine [94].
- The essential role of health systems in technology exchange to nationalize Covid-19 vaccines in the Global South [95].
- Co-production of knowledge by public health agents, experts, and US and Brazilian patients, on the topic of Long Covid [96].

These case studies illustrate diverse contributions of emerging economy health systems to the advancement of biomedical research and health technologies. At the same time they demonstrate the reactive nature of health systems, which tend to respond to local health issues and crises, rather than proactively developing long-term efforts to align institutional readiness with the evolving R&D landscape to address health challenges [97].

#### Ethics, transparency and democratic values

Effective democratic policies for funding R&D activity are critical in advancing emerging technologies. Confidence in ethics committees, pharmaceutical agencies, and regulatory bodies is essential. Scholars have noted that the absence of well-defined regulations and democratic institutions capable of addressing issues in technology development, animal experimentation, and clinical trials is a primary challenge faced by scientists and developers seeking to collaborate with emerging economies [98].

Respect for regulations has historically been institutionalized as part of the routine of knowledge production in biomedical domains, a concern for researchers from the early stages of technology development. In nascent fields such as molecular systems engineering, regulatory limitations are even capable of redirecting research agendas. In Europe and the United States, clear-cut guidelines and regulatory bodies composed of science and bioethics experts are understood as essential to impartial examination of ethical concerns [99].

AE in clinical innovation introduces a new level of complexity, as knowledge on engineering, computing and mathematics operate in different regimes of norms and regulations, with a traditional distancing from animal subjects, or biological or living things. Additionally, ethical and regulatory considerations of STEM research differ substantially from biomedical research and clinical interventions. For example, how will scientists conducting AI-assisted nanomaterials discovery assure ethics committees composed of health professionals that the potential risks of autonomously-synthetized chemicals have been anticipated and accounted for? This is also a concern in well-established health research organizations.

If ethics and transparency are critical, this debate must advance to the level of public exchange. Lack of transparency in reforming institutions for AI and other digital transformations in health-related research can have unintended results, in some cases damaging societal sympathy towards new technologies. Are democratic regimes in emerging economies prepared to provide an arena for discussion of this technological transition marked by intense convergence of STEM knowledge into healthcare [100, 101]?.

Cases from India [102], China [103], the Philippines [104], and Iran [105] demonstrate how a lack of democratic policies can restrict meaningful research collaboration at critical stages, due to high levels of uncertainty or imprecisely defined tech regulation. Integration of AI into the healthcare sector presents a challenge for both developed and emerging economies, as both regulatory and scientific communities are still establishing consensus and rules in this field. Reform in legal frameworks will be critical for coordination between AE developers and emerging economy stakeholders.

### Opportunities

AI present stakeholders in emerging economies with a range of new opportunities [106]. In this section I highlight six of these areas.

#### Local expertise in digital health technologies

The AE community may lack awareness of experts in emerging economies, and their potential as collaborators. For decades, engineer scientists from emerging economies have developed tools and technologies in the fields of bioinformatics, computation, and automation with high levels of success [107, 108].

I would like to highlight two examples from India and Brazil, regarding laboratory autonomation and AI-assisted systems in healthcare. In India, the 2017 launch of Aptio Automation, the first fully automated track lab, brought automation lab innovation in the country to a new level. This initiative involved years of multidisciplinary research and robust investments from local companies and industry leaders [109], fostering a partnership between science, manufacturing, hardware and software experts [110]. Capabilities held in those projects work as a set of fundamental knowledge which could allow stakeholders to develop AE systems locally [111].

In recent years emerging economy researchers have opened avenues for collaboration, merging competencies towards constructive interface between healthcare and AI-driven knowledge platforms. For example, new capabilities developed in Latin America are fundamental to improving data robustness and to feed generative-AI integration into healthcare innovations. A recent project in Brazil well-successfully interfaced technical skills between automation systems for a mega volume reference clinical laboratory, creating an interconnected system capable of linking nearly one hundred different analyzers and seven clinical specialties [112].

Integration among scientific, engineering, and health research competencies are needed to propel AE towards clinical application. But this translational work should not be taken for granted. In AE’s current stage, developers are actively designing and prototyping efficient, precise, and reproducible systems, while partners from the healthcare sector serve as co-developers [113]. International collaborations producing large amount of clinical data serve as robust input to AE R&D hubs, and they might benefit from exchange with innovators from emerging economies.

#### Reducing disadvantages through digital collaboration

S&T policies and research institutions from emerging economies face disadvantages compared with high-income countries [114]. To foster AE globally, decentralized digital platforms based in robust human-computer collaborations can serve as strategic infrastructure to support health innovation.

Initiatives abound in southeast Asia, with meaningful knowledge collaborations happening in basic research in areas such as chemistry, biophysics, computation, and materials sciences [115]. The Asian Consortium of Computational Materials Sciences (ACCMS), as an example, engages researchers from Japan, India, China, Taiwan, Malaysia and other nations. Stakeholders from Singapore, a high-income country which plays a key role in fostering qualified regional knowledge networks in health technologies in eastern Asia, lead the joint labs of the Advanced Remanufacturing and Technology Centre (ARTC), launched by the Agency for Science, Technology and Research (A*STAR) in partnership with Nanyang Technological University of Singapore [116, 117]. This lab is noteworthy for its success in gathering private sector stakeholders from digital health, data-intensive biotechnology research, and AI-assisted materials and drug discovery [118].

As examples of North–South collaboration, the Vector Institute of Artificial Intelligence in Toronto, Canada promotes the international exchange of scholars, students and private sector professionals with countries like Mexico, India and South Africa [119]. Tecnologias de la Informacion y Comunicacion of the Programa Iberoamericano de Ciencia y Tecnología para el Desarollo, between Spain and partners in Latin America, executes strategic projects on automation [120]. Finally, the SDL tool Polybot is a bio-inspired microelectronic tool that combines AI and robotics to speed discovery of wearable biomedical devices. Polybot is housed in the Argonne National Laboratory in Lemont, Illinois, and will be soon open to international scholars [121]. Such partnerships between regions could support foreign stakeholders in overcoming barriers to scientific progress.

#### Artificial intelligence to address global health issues

The way drug discovery systems are organized and funded has so far proven incapable of solving many persistent health issues worldwide. Present systems of science and technology provide few models to challenge the status quo or privilege knowledge generated outside the Global North [122, 123]. Accelerating AE for clinical innovation is of great interest for public health in emerging economies, where stakeholders can utilize AE systems to address global health issues relevant to their own context.

Health emergencies require comprehensive societal coordination in any setting. The Covid-19 pandemic, as an example, proved to be an even greater challenge in global south [124, 125], further evidence of the opportunity presented by decentralized AE collaborations for global health challenges.

AE can have important impacts in emerging economies in areas like vaccine development for neglected diseases and re-emergent epidemics [126], and molecular diagnostics and precision oncology tools for cancer patients. But how? Emerging economies are centers of neglected and tropical disease knowledge due to the social and political relevance of these conditions. Countries like India, Brazil, Taiwan, South Korea and Indonesia are potential strategic partners for international AE consortia in these areas, due to their capacity in vaccine R&D, public health policy, systems, and planning. The healthcare innovation sector in these nations can contribute to addressing challenging tropical diseases, epidemics, and their social impacts in local communities.

#### Setting a science and innovation diplomacy agenda

The relatively recent movement of science and innovation diplomacy (S&ID) aims at fostering exchange of technical and political capabilities among individuals governing science, technology, and innovation systems and foreign policy. It has proven a useful tool for emerging economies to take part in international networks of scientific collaboration [127]. S&ID has evolved rapidly in emerging economies, resulting in knowledge production, local and international initiatives, and implementation of multilateral forums (with several currently under institutionalization) to approximate science and innovation competencies from foreign policy bureaucrats [128, 129].

S&ID employs existing expertise and established foreign policy knowledge infrastructure to promote scientific and technological collaboration, presenting an opportunity for emerging economies. A diplomatic approach can mitigate differences between disciplines and expertise in favor of common interests, helping direct political attention to the value of AE for health discovery and innovation.

S&ID has been utilized by international organizations to promote equitable health innovation agendas in emerging economies. Working groups at the Pan American Health Organization (PAHO), the Global Alliance for Vaccine and Immunization (GAVI), and the Organization of American States’s Inter-American Committee on Science and Technology (COMCyT) have been integral to supporting scientific and technological collaborations aligned with the priorities of individual national healthcare systems.

As bureaucrats tend to demand quick responses to short-term tasks, diplomats and politicians may not be fully prepared to respond to scientists’ priorities and relentless dedication to advancing the frontiers of their field with colleagues and peers [130]. Similarly, scientists may not be concerned with the political dividends of their collaborations [131]. To be effective, S&ID initiatives addressing AE must find ways to attract the participation of scientists, and provide adequate training to policy experts on how to manage programs for innovation in health technology.

#### Co-producing the ethical and regulatory landscape

AE is still in its early years, with significant differences in ethical and regulatory landscapes between countries. Also, there are many institutional voids to address. While coordinating among scientists, governments, industry, clinicians, and regulators is not an easy exercise, emerging economies can seize this opportunity to co-produce useful ethical guidelines and regulations for AI in biomedical research and in the healthcare sector. In ensuring inclusion of emerging economies, we can establish frameworks for ethical guidelines, governance, and regulatory standards for responsible uses of AE that reflect a broader range of perspectives and priorities. As is the case for many early stage technologies, AE developments in health-related domains may create uncertainty among researchers and society regarding how beneficial AI interventions in biomedicine actually is, as AI-assisted drug discovery or nanomedicine for example. Partnerships among the community of AE scientists and developers can catalyze the co-production of a suitable ethical and regulatory landscape.

Scholars have advanced the debate on the ethical and regulatory aspects of AI and digital technologies in healthcare. Gwagwa and colleagues (2019) criticize AI as a panacea for mitigation of inequities in many African societies, noting that “both the benefits and risks of AI are readily apparent” [132]. Alami et al. (2020) explore how to make AI in healthcare more responsible, sustainable, and inclusive in emerging economies [133]. Likewise, studies have illustrated the significant challenges faced by governments and healthcare systems in utilizing knowledge infrastructures to address public needs – underscoring the paradox between the level of sophistication of biotechnologies apparently available for all, and the lack of resources present in emerging economies to fully participate [134].

AE is unique in that it involves deeper philosophical and societal considerations about how science is defined, and how science and technology are produced [135]. AE opens possibilities for hypothesis generation and data-feasibility of projects, altering the traditional inductive nature of scientific research - in which a problem is followed by a literature review to formulate a question, which then guides the construction of a method, and finally testing to achieve results. Since AE experts see this model as inefficient, building robust platforms capable of running experiments autonomously, and aiming to accelerate scientific discovery, requires broader public debate regarding its implications to society [136].

Until the present, AE development has adhered to existing research ethics guidelines and regulations. As societal awareness of AE grows, novel ethical questions and regulatory considerations can be expected. More empirical research is needed to support the creation of effective ethical guidelines and policy recommendations for AE innovation. Due to the novelty of AE in science and medicine, it can benefit from international collaboration concerning ethical aspects and societal impacts.

#### Diversity, equity, inclusion, and trustworthiness (DEIT)

It is imperative that stakeholders promote diversity, equity, inclusion, and trustworthiness (DEIT) in the field of AE. Active involvement of emerging economies in development and implementation is key to wider dissemination of this technology. An inclusive approach, as applied in other STEM research fields, supports equitable technological advancement [137].

Diversity refers to a range of geographic, cultural, and socioeconomic features. AE benefits from the experiences and expertise of emerging economy researchers who might be off the radar of leading institution researchers. Their inclusion leads to more comprehensive research outcomes, as different regions face unique circumstances that can inform the development of AE.

The values of equity and inclusion reinforce the importance of equal opportunity for all stakeholders in the SDLs initiative. Global research efforts should prioritize partnerships that offer capacity building, technology transfer, and financial support, to promote active participation and meaningful contribution by lower-income regions. Democratizing access to SDLs and MAPs, and sharing knowledge, can empower local entrepreneurs to develop solutions for their specific context [138]. AE will generate higher levels of creativity with an inclusive approach, as other science and innovation fields have found in recent years [139].

Trust in emerging science and technology is understood to be critical for healthcare innovation. In its absence, the effects on technology can be profound, as we have seen in cases of unproven biotechnologies, such as stem cell research in China and Japan [140, 141]. Ethical and responsible use of autonomous technologies is crucial for cultivating trust in society and among all stakeholders.

To facilitate a DEIT approach in the area of AE, international organizations, governments, and private sector stakeholders must act together. Promoting DEIT in global AE research is an ethical imperative, but also a strategic advantage. Collaborative funding mechanisms, technology-sharing agreements, and knowledge exchange platforms can all pave the way for meaningful participation.

## Conclusions

The potential of AI in biomedical research and health innovation are yet to be realized. As these technologies continue to advance, we can expect further breakthroughs in R&D and clinical innovation, ultimately leading to improved health outcomes.

AE presents an opportunity for stakeholders from emerging economies to co-produce the global landscape of AI in biomedical sciences and health innovation. However, an attentive sociological analysis should acknowledge asymmetries in R&D capabilities among countries, since emerging economies suffers from inadequacies and discontinuities in resources and funding. Early consideration about those issues by policymakers and investors can accelerate the design and implementation of policies and programs in emerging economies aiming to increase the presence of global south stakeholders in the emerging field of AE. It could shed light to new opportunities and agendas that emerging economies are well positioned to play, as AI applications to solve global health issues, AE to accelerate the biopharmaceutical development and solutions to high-prevalence diseases as cancer, AI to improve quality of collaborations in clinical studies, and so on.

By actively involving emerging economies in this transformative field, stakeholders involved with AI in the sciences produce a more equitable and robust science and technology landscape. The establishment of decentralized AE infrastructures and initiatives could overcome local restrictions, fostering ongoing capabilities in emerging economies, and open broader venues for a more culturally diverse innovation environment for the growth of the field. Additionally, promoting an equitable, inclusive and trustworthy development of AI in health-related research and innovation domains could facilitate the building of meaningful partnerships and engagement. By improving the geographical representativeness of AE, emerging economies contribute to facilitate the diffusion and acceptance of AI in health-related R&D internationally. Through collaboration and inclusivity, we come closer to realizing the potential of AE to solve global science and health challenges.

A social and political analysis of AI implications in health innovation, in general, and of AE interventions in biomedical research, specifically, could help strengthen AI to enhance biomedical knowledge infrastructures worldwide, led by values such as trustworthiness and equitable access to allow researchers to address health issues of global interest and public impact. Improving institutional preparedness in emerging countries is critical and could enable stakeholders to navigate opportunities of AI in biomedical research and health innovation in the coming years.

## Data Availability

Data used in this study can be accessed by demand through emailing the author.

## Declarations

### Authors’ information

Renan GL da Silva is a postdoctoral researcher in the Health Ethics and Policy Lab at the Department of Health Sciences and Technology, ETH Zurich, Zurich, Switzerland. His research focuses on the social, ethical, and political issues related to the introduction of emerging technologies in biomedical research and innovation in multiple organizational settings. Recently, da Silva is dedicated to the empirical study of practices and interventions driving the expert knowledge production in Bioengineering-related domains (e.g., Molecular Systems Engineering), responsive bionanomaterials, self-driving labs and precision medicine.

### Sex and Gender Considerations

The author applied general principles present in the Sex and Gender Equity in Research (SAGER) Guideline [142] for reporting of sex and gender information in all versions of the manuscript concept and design, literature review, and interpretation of data. Since this is a debate article, no empirical data collection and analysis was performed. However, attention was dedicated to provide fair representation of gender, race and ethnicity in the selection of studies to be discussed. As a Latino Scholar and first generation academic in my family, I have experienced many situations in which ethnic and gender issues in academic environment has been completely gaslighted. Then, I valuate and appreciate such initiative that might shed light to this issue among the team of authors and reviewers of this respected publication.

## Abbreviations

A*STAR: Agency for Science Technology and Research
ACCMS: Asian Consortium of Computational Materials Sciences
AE: Autonomous experimentation systems
AI: Artificial Intelligence
AMPs: Acceleration Materials Platforms
ARPA H: Advanced Research Projects Agency for Health
ARTC: Advanced Remanufacturing and Technology Centre
CEE: Center for Excellence in Education
COMCyT: Organization of American States’s Inter-American Committee of Science and Technology
CYTED: Programa Iberoamericano de Ciencia y Tecnología para el Desarollo
DARPA: Defense Advanced Research Projects Agency
DENDRAL: DENDRAL Project
DoE: Department of Energy of the United States
GAVI: Global Alliance on Vaccine and Immunization
KAIST: Korean Advanced Institute of Science and Technology
ML: Machine Learning
PAHO: Pan-American Health Organization
R&D: Research and Development
S&ID: Science and Innovation Diplomacy
S&T: Science and Technology
SDLs: Self-driving Laboratories
STEM: Science Technology Engineering and Mathematics
